# On the Search for Grazing Personalities: From Individual to Collective Behaviors

**DOI:** 10.3389/fvets.2020.00074

**Published:** 2020-02-25

**Authors:** Cristian A. Moreno García, Thomas M. R. Maxwell, Jonathan Hickford, Pablo Gregorini

**Affiliations:** Department of Agricultural Sciences, Faculty of Agriculture and Life Sciences, Lincoln University, Christchurch, New Zealand

**Keywords:** grazing patterns, behavioral syndromes, conceptual model, genotype-to-phenotype associations, heritability, social environment, personality plasticity

## Abstract

While grazing lands can offer a diverse range of forages, individuals within herds prefer to graze some habitats and not others. They can have consistent differences in grazing patterns and occupy specific spatial domains, whilst developing tactics and strategies for foraging that are specific to their grazing personalities. In this review, we explore the development of our understanding of grazing personalities, as we move away from the search for an “optimal animal” toward designing behavior-customized herds with an arrangement of individual grazing personalities that enhance ecosystem services and productivity. We present a “grazing personality model” that accounts for the personality of individual animals and for collective behaviors of herds. We argue that grazing personalities of grazing ruminants and other large herbivores are in part genetically determined, and that they can act at the individual and collective level. The social and biophysical environments as well as the emotional state of animals regulate the expression of “grazing genes” that are observed phenotypically as distinct grazing personalities. The reproductive and sexual successes of individuals and herds filter for allele variants of grazing genes and in turn determines their relative frequency. While the selection of one grazing personality may be adequate for homogeneous pastoral systems, the design of herds with a range of grazing personalities that are matched to the habitat diversity may be a better approach to improving the distribution of grazing animals, enhancing ecosystem services, and maximizing productivity.

## Introduction

We picture foraging animals distributed throughout grazing lands. Individually or in various sized groups of one or more species, herbivores explore and graze a diverse range of habitats including riparian areas, open flat plains, gentle or steep hills and mountainous lands. Even when considering herds of one single species, individuals show divergent dietary tactics and foraging site preferences resulting in consistently and regularly repeated grazing patterns, like for example in cattle or sheep (1–4).

Grazing has been described as a process composed of short-term ingestive tactics, and mid- and long-term digestive strategies (5), and its pattern is defined as a cluster of decisions that lead to ingestive actions and digestive strategies that are motivated by the interaction of both internal and external stimuli (6). Differences in grazing patterns are far from being trivial or random, with individual animals behaving consistently and adopting specific grazing strategies across situations and over time; such that animals are said to display recognizable grazing personalities (7–10). The diversity of grazing personalities within herds modulates the intensity and frequency of forage defoliation achieved with recommended stocking rates, the fitness of animals and other production traits such as reproduction success, survival, and live-weight changes (11, 12). As a consequence, differences in grazing patterns and personalities affect ecosystem functions such as speeding up nutrient cycling (13), increasing productivity of grasslands (14), and preventing loss of plant diversity (15).

The concept of animal personality, also referred to as behavioral syndrome (10), copying style (16), and temperament (17) among other closely related terms (18, 19), was developed by integrating correlated traits of behavior with other traits. For example, Carere and Maestripieri (20) defined animal personality as correlated behavioral and physiological traits that differ among individuals of the same species, and that are temporally stable across different contexts or situations. Gosling and John (21) suggested this concept should not be restricted to differences observed within-species, but rather these are behaviors and patterns that are consistently displayed by individuals regardless of the species identity. Some authors also argue that personality should include traits that account for consistent patterns of feelings and thoughts that affect behavior (22). In this way, the concept of animal personality includes emotional and cognitive traits, which can influence animal decision-making and well-being. In line with Maderspacher's (23) arguments and Biro and Post's (24) speculations, we have chosen to include morphological traits in our definition of grazing personality, as evidence showed correspondence between behavioral polymorphisms and morphological polymorphisms. Accordingly, we define grazing personality for grazing ruminants and other large herbivores as “suites of traits of different nature (e.g., behavioral, cognitive, physiological, and morphological), which are correlated and often concatenated, to result in specific grazing patterns displayed consistently across contexts and over time.”

Regardless of the species identity, differences in grazing personalities are observed at the individual (8, 21, 25, 26) and collective level; that is in groups, herds, and populations of animals (27, 28). Consequently, we argue that grazing personalities are the result of evolutionary processes that filtered alleles and established allele frequencies of key genes related to behavioral patterns, tactics, strategies, and decision-making in the grazing process, hereafter referred to as “grazing genes.” In addition, interactions with social and biophysical environments, the emotional state of animals and their experiences early in life, might modify the epigenome of grazing genes, thereby modulating their expression.

We support the contention that grazing personalities are observable at individual and collective levels, and suggest that divergent grazing personalities result in distinct grazing patterns and attributes; such as the ability to explore, define a home-range, display a habitat preference, and fragment into groups. These all affect the ecological functioning of grazing systems.

We also propose a “grazing personality model” (GP-model). The purpose and context of the GP-model is to represent the genetic elements, the regulatory systems, and the phenotypic elements that encompass individual and collective personalities in a context of herds of grazing ruminants and other large herbivores. The objective of the GP-model is to further develop our understanding of distribution of grazing animals following the initial “Ecological-Hierarchical grazing model” (29) and the additional concepts of the “Distribution Patterns and Mechanisms” model (30). The GP-model represents grazing personalities, which are genetically determined (genotypic personality) and epigenetically modulated through systems that regulate the expression of grazing genes (personality plasticity) via interactions with the social herd environment and the biophysical features of the grazing environment. The emotional state of animals influences the regulatory systems that modulate gene expression and affects grazing decision-making. In this review, we first deal with grazing personalities at the level of the individual animal, then we deal with collective personalities and finally we illustrate GP-model implications based on movement ecology, genetics and animal personality.

## Individual Grazing Personalities

“*…from the population optimum perspective […] natural and sexual selection may favor the evolution of multiple responses to environmental challenges, thus resulting in within-population variation in the same behavioral trait, and in whole suites of behavioral traits”* (31).

**Figure 1:**
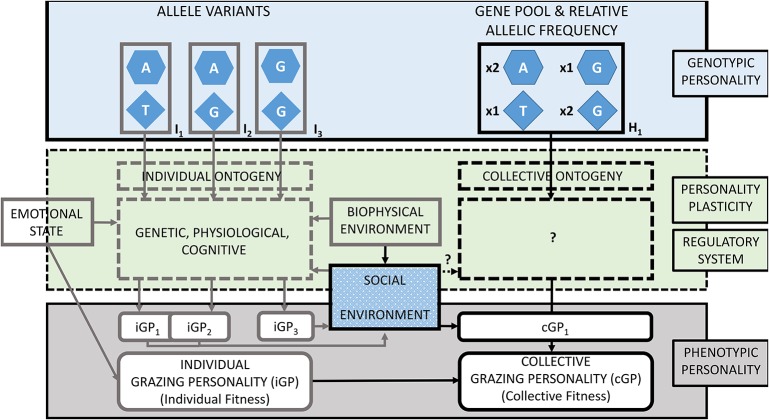
The Grazing Personality Model (GP-model) for ruminants and other large herbivores described by three main aspects: the genotypic personality (top), regulatory system conferring personality plasticity (middle), and the phenotypic personality. In the example, three hypothetical combinations of allele variants (I_1_, I_2_, and I_3_) applied to two grazing genes represented at the individual level (left side). The genotype of individuals constitutes the gene pool and the relative allelic frequency (H_1_) of grazing genes at the collective level (right side). Individual and collective grazing personalities (iGP and cGP) are phenotypically represented with corresponding fitness. The example shows two grazing genes in beef cattle (32): the glutamate receptor 5 in chromosome 29 (hexagons) and the mastermind-like 3 in chromosome 17 (diamonds). Allele variants specified by nucleobases adenine [A], guanine [G], and thymine [T]. The interactions between genes and environment regulates the expression of grazing genes and confers personality plasticity. Phenotypic grazing personalities of individuals (iGPs) may overlap (e.g., iGP_1_ and iGP_2_) or diverge (e.g., iGP_3_). A group of individuals coexisting and displaying distinct grazing personalities constitutes a grazing herd with its own collective grazing personality (cGP). Adapted from Bengston and Jandt (33); with concepts from Koolhaas and van Reenen (34), Robinson (35), and Sih (10).

This section describes the GP-model at the individual level (Figure 1, left side): from individual genotypes of grazing personalities at the top, through regulatory systems that modulate the gene expression and confers the personality plasticity in the middle, to individual phenotypic grazing personalities at the bottom. Thus, in section The Genetics of Behavior and Grazing Related Genes we present evidence about grazing genes and its heritability. We then investigate regulatory systems that modulate the expression of grazing genes in variable responses to stimuli conferring the personality plasticity (see section The Effect of Personality Plasticity and Regulatory Systems on Grazing Patterns). Finally, in section Grazing Traits of Individuals we present examples of phenotypic grazing personalities and traits at the individual level (Table 1).

**Table 1:** Dichotomous and multiple classifications of animal behavioral types in grazing ruminants and other large herbivores.

**Species**	**Behavioral types**	**Behavioral categorizing criteria**	**Continuous and categorical variables**	**Genetically explained**	**References**
Beef cattle	1. Riparian areas users 2. Uplands users	1. Home-range fidelity	Categorical (dichotomous) and continuous	Probably	(2, 36)
Beef cattle	Breeds better suited for mountainous terrain	1. Slope 2. Horizontal distance 3. Vertical distance	Continuous	Probably	(37)
Beef cattle	1. Bottom dweller 2. Hill climber	1. Terrain-use indexes	Continuous	Probably	(1)
Beef cattle	1. Dominant 2. Subordinate	1. Dominance	Continuous	No	(38)
Beef cattle	1. Fast-eater 2. Slow-eater	1. Supplement intake rate	Continuous	Probably	(39)
Beef cattle	1. Bottom dweller 2. Hill climber	1. Terrain-use indexes	Continuous	Yes	(32)
Beef cattle	1. Bottom dweller (?) 2. Hill climber (?)	1. Terrain-use indexes	Continuous	Yes	(40–42)
Beef cattle	1. Favorable distribution 2. Unfavorable distribution	1. Terrain-use indexes (?)	Categorical (dichotomous) and continuous?	Yes	(43)
Beef cattle	1. Highly exploratory/bold 2. Slow-exploratory/shy	1. Response to novel object	Categorical (dichotomous) and continuous		(44)
Dairy cattle	1. Low residual 2. High-residual	1. Residual feed intake	Continuous	Yes	(9)
Highland beef cattle	1. Initiator 2. Follower	1. Leadership 2. Dominance	Continuous		(45)
Multiple species (mice, rats)	1. High-aggressive 2. Low (medium)-aggressive	1. Aggressiveness	Categorical (dichotomous) and continuous	Yes	(46)
Multiple species (foragers).	1. Leader 2. Trailer A.Speeder B.Laggards	1. Walking speed 2. Accelerations to conspecifics 3. Length of decision zones 4. Sense of orientation	Categorical (dichotomous) and continuous		(47)
Sheep	None specified	1. Sagebush consumption/dietary selection	Continuous	Yes	(3)
Sheep	1. Bold 2. Shy	1. Shyness-boldness	Categorical (dichotomous) and continuous		(48)
Sheep	1. Bold 2. Shy	1. Shyness-boldness	Categorical (dichotomous) and continuous		(49)
Beef cattle	None specified	Consumption of several species of grasses and forbs	Continuous	Yes	(4)
Beef cattle (Nellore)	None specified	1. Crush score 2. Flight speed 3. Movement score 4. Temperament score	Categorical (nominal) and continuous	Yes	(11)
Deer	Several combinations of multiple dimensions	1. Boldness 2. Dominance 3. Flexibility	Categorical (nominal) and continuous		(50)
Multiple species (foragers) with whole spectrum of personality types	1. Superficial explorer/bold/aggressive 2. Thorough explorer/shy/ non-aggressive	1. Exploration strategy 2. Boldness 3. Aggressiveness	Categorical (dichotomous) and continuous	Yes	(51)
Multiple species (cattle, horses, pigs)	1. Proactive/bold 2. Reactive/docile 3. 15 combinations of three-dimensions personalities	1. Coping style 2. Emotionality 3. Sociality	Categorical (dichotomous and nominal) and continuous	Yes	(34)
Multiple species (foragers) with whole spectrum of personality types	1. Fast-explorer 2. Slow-explorer	1. Area-restricted search (fractal movement) 2. Sense of direction 3. Home range size and structure 4. Aggressiveness	Categorical (dichotomous) and continuous		(52)
Multiple species (African elephant, Galapagos tortoises, mule deer)	1. Central place foraging 2. Migration 3. Nomadism	1. Node-level (local) metrics 2. Graph-level (system) metrics	Continuous		(27)

*See Réale et al. (19) and van Oers and Sinn (53) for studies with genetically-associated behaviors; see Smith and Blumstein (12) for single personality dimension related to fitness. Behavioral types, behavioral categorizing criteria, type of variable, and if behavior has been explained genetically*.

Individual animals exhibit repeatable differences in their grazing behavior within populations, within species and across species. These personality differences arise for many reasons, such as differences in permanent environmental effects (e.g., familial, parental, and epigenetic contributions) and the effect of genetic variation. In ruminants, personality differences can influence eating tactics and ingestive behaviors (9, 54). For example, Gregorini et al. (9) studied a group of 16 dairy cows that were selected as calves (6–8 months old) based on divergence in residual feed intake (i.e., having high and low residual feed intakes) and measured their individual grazing behaviors, eating patterns, and ingestion tactics as milking cows. From a grazing behavior viewpoint, low residual feed intake individuals prioritized grazing and ruminating over idling. They typically took fewer steps when walking during grazing and had a higher ratio of grazing to non-grazing steps when compared with cows with high residual feed intake. From an ingestive viewpoint, low residual feed intake individuals masticated less, but ruminated more intensively, and they had feces with 30% less quantity of large particles size than their counterparts with high residual feed intake. Wesley et al. (39) also pre-classified 18 beef cattle heifers from within 80 animals in two consecutive years (*n* = 36) based on the rate of consumption of supplementary feed (another trait related to the eating tactics). Similarly, the authors reported divergent grazing behavior and ingestion tactics; for example, cows with faster rates of consumption of supplement tended to spend less time at water, cover larger areas and exhibit less concentrated grazing search patterns than cows with slower consumption rates. These two studies speculated a link between the divergent phenotypic behaviors (i.e., eating tactics) displayed by the selected animals and their genotype. In the following section, we present the genetics of behavior and genes related to grazing patterns.

### The Genetics of Behavior and Grazing Related Genes

Van Oers and Sinn (53) undertook a meta-analysis of studies on animal personality to quantify the heritability of personality in wild, captive and domesticated populations of a range of animals. The statistical meta-analysis included 209 estimates of heritability on 14 taxonomic groups such as Ruminantia, Equidae, Canidae, and Hymenoptera, just to mention a few. The authors reported an average heritability of 0.26 for animal personality traits with a cumulative size effect (*E* = 0.18) significantly different from zero. The average heritability was higher in wild populations than in domesticated populations (0.36 > 0.24), and unweighted heritability estimates for exploration behavior were 0.58 and 0.21, respectively. These authors concluded that selection of animals based on their personality could be expected in wild populations.

At a more general level, a more recent meta-analysis of behavioral studies on non-human animals reported estimates of heritability and repeatability of animal personalities (55). After screening 306 relevant articles, they selected 10 research studies and 71 pairs of estimates for analysis. Their analyses suggested that the repeatability of behavioral responses has a substantive genetic component, with the study revealing that 52% of the phenotypic variation in general behaviors such as aggression, antipredator, foraging, parental effort, and mating, was attributable to additive genetic variation (i.e., genotypic personality in the GP-model). The authors also reported a greater and large mean heritability for animal personality (0.52) than for behavioral variation (0.14). Animal personality heritability being inclusive of additive genetic variation, dominance genetic variation and permanent environmental effects, while behavioral variation includes in addition the temporal environmental effects. If genetic dominance (i.e., non-additive genetics) plays a minor role in determining animal personality (55), then one can potentially attribute about half of personality variation to the effects of the social and biophysical environment (e.g., parental care and vegetation characteristics, respectively) and to epigenetics. In the GP-model, these effects are referred to as the regulatory systems and the personality plasticity. However, it remains unclear how much of the non-additive genetics [i.e., allelic interactions at the same locus (dominance) or at different loci (epistasis)] can explain the phenotypic behavioral variation (56). For example, in humans, non-additive effects could be as significant as the additive effects in explaining several dimensions of personalities (57).

Results of Dochtermann's et al. (55) meta-analysis are promising but provisional and need to be taken with caution. They also reported that foraging behaviors had a much weaker genetic component (<0.2) than aggression and antipredator behaviors (up to 0.6).

Recent studies supported the premise of grazing personality being under genetic control. Howery and Bailey (43) described both genome regions and gene markers associated with grazing distribution patterns in beef cattle. As an example, using collared cows (*n* = 87) that carried global positioning systems (GPS), Bailey et al. (32) investigated the association of several quantitative trait locus (QTL) and genetic markers with the phenotypic variation of grazing patterns of cattle displayed along gradients of steep-sloping terrain, elevation and distance to water sources. These cows were grazed in mountainous and extensive grasslands at five ranches in New Mexico, Arizona and Montana in the United States of America (USA). A high-density single nucleotide polymorphism (HD SNP) array was used to genotype DNA samples from these cows. The study then ascertained whether associations existed between variation in the SNP markers and variation in grazing distribution based on indexes of terrain use. Two QTLs overlaying the glutamate receptor 5 (GMR5) gene accounted for up to 24% of the phenotypic variation in the use of vegetation patches on steep slopes and at high elevations, while another QTL overlaying the mastermind-like 3 (MAML3) gene accounted for 23% of the phenotypic variation (Figure 1). These genes have been reported to be involved in locomotion, motivation, and spatial memory as well as in the regulation of neurogenesis, myogenesis, vasculogenesis, and other aspects of organogenesis.

Studies conducted by Pierce et al. (40, 41) validated the previously reported genotype-to-phenotype associations between specific SNPs overlaying grazing genes and indexes of terrain use (32). While these results are promising and point toward the possible integration of grazing personality into selection programs, Howery and Bailey (43) suggested these studies need to be replicated and/or extended to larger number of animals of different origin and which are grazed in diverse environments, if robust and conclusive conclusions are to be reached. For example, the extended study of Pierce et al. (42) including 330 beef cows from 14 ranches in the western USA reported limited genotype-to-phenotype associations and pointed toward different candidate genes.

There are two outstanding explanations for the correlation of behavioral traits defining grazing personalities. The first one is pleiotropy, in which one gene could act on two or more traits, which further determine the displayed grazing patterns. If pleiotropy occurs, one single gene would effectively control several traits simultaneously. For example, phenotypic studies corroborated the correlation of distinctive grazing patterns (e.g., fast-explorer cows), growth rates and boldness within relatively small groups of cattle [i.e., 16 and 36 individuals in Gregorini et al. (9) and Wesley et al. (39), respectively].

Kern et al. (58) suggested that pleiotropic effects could explain the correlations between personality, morphological and performance traits on zebrafish (*Danio rerio*), but also did not confirm this possibility. A study with bighorn sheep from Ram Mountain, Alberta, Canada could not find pleiotropic effects at major locus because of the lack of genome-wide QTL overlap on genes related to docility and boldness (59). Instead, the authors concluded that small pleiotropic effects could have been missed and therefore, results did not confirm pleiotropy. Future studies might give insights of pleiotropy controlling grazing personality traits.

The second explanation for the correlation of traits as observed in grazing personalities is because of a non-random association of alleles at different loci that produce a combination of traits that confers advantage under a specific set of biophysical and social conditions, as is the case of linkage disequilibrium. For example, individuals with certain association of alleles tend to achieve higher reproduction rates than individuals with a different combination of traits. Such allele associations become common and more frequent in a population than other combinations, although traits are controlled by alleles at different loci (53).

Studies suggested that genetic variation might explain different eating tactics linked to distinctive grazing behaviors exhibited in groups of beef heifers (39) and dairy cows (9). For the latter example, Davis et al. (60) previously confirmed the different genetic basis found on nearly 200 dairy cows that, within a large herd of 3,359 milking cows, displayed extreme residual feed intakes. These genetically tested cows were mother dams of the 16 calves used on Gregorini's et al. (9) research. Future research on grazing personalities and its genetic variation might help to elucidate whether grazing traits are correlated because of genetic pleiotropy, or because of a linkage disequilibrium between grazing traits, or because of both mechanisms acting simultaneously. Both, pleiotropy mechanisms as well as linkage disequilibrium were represented in the hierarchical conceptual model “Organization of Behavioral Traits” (19) and have implications for the regulation and expression of grazing personalities.

The discovery of genetic associations with grazing personalities and thus the identification of specific grazing genes has the potential to assist in breeding programs. However, despite the high heritability of grazing patterns found in cattle, there are other factors controlling them. For example, interactions with the social herd environment (e.g., parental and familial effects), the biophysical environment, and the emotional state as well as the large number of range management practices that influences such interactions. In the next section, we discuss whether non-genetic factors can modulate the expression of grazing genes and if such effects over gene expression are transferable to offspring.

### The Effect of Personality Plasticity and Regulatory Systems on Grazing Patterns

The section The Genetics of Behavior and Grazing Related Genes and the section Gene Pools and Allele Frequencies focused on alleles of grazing genes, their variation and frequency at two levels, individual and collective, respectively. In the GP-model, allele attributes of grazing genes are the ultimate determinants of grazing personalities. These attributes constitute the individual and collective genomes, respectively, and account for the specific sequence of nucleobases of each gene; that is the genome code. The gene products expressed into RNA and subsequent amino acids and proteins are the ones executing the observed phenotypic traits, such as behavioral traits. In this section, we focus on gene expression and regulatory systems that modulate the expression of behavioral genes related to grazing personalities. Here, we present the ontogeny, the epigenetic inheritance system, and the animal emotional state as the main modulators of behavioral gene expression. These three components of the GP-model create the interface between the genomic determination of grazing personalities and the external and internal stimuli that modulate its gene expression. The expression of grazing genes is variable and responds to changing environmental conditions and emotional states; regulatory systems modulating the gene expression and thus conferring the personality plasticity of the GP-model.

Regulatory systems are an integral part of the pathways between grazing genes and the observed grazing personalities. In the GP-model, grazing personality pathways originate from specific alleles of grazing genes and result in specific phenotypic grazing patterns. Grazing personality pathways involve hierarchical levels of intermediate and concatenated traits with multiple mechanisms that consistently respond to external and internal stimuli modulating the observed grazing patterns. The “Organization of Behavioral Traits” (19) conceptualized genes-neurophysiology-behavioral pathways in a hierarchical model where a few genes are involved in determining a few neurological, physiological, and morphological traits. These neurological, physiological and morphological traits further shape the expression of a number of behavioral traits that ultimately result in biological functions, such as herbivore grazing patterns. As the gene expression of intermediate traits is variable in response to stimuli, each adjusted response of intermediate traits is added up and further transferred along pathways of grazing personality (19).

The variable expression of grazing genes modulated by regulatory systems is referred to as grazing personality plasticity. The reaction norm of behavioral traits are examples of behavioral trait plasticity changing along environmental gradients (61). As the phenotypic response along environmental gradients differs from one individual to another, the grazing personality plasticity might be a trait by itself and even have its own heritability (62). However, even if environmental conditions stay unchanged, the behavior of an individual changes as it ages, which is known as ontogeny, and that leads to behavioral development (63).

#### Ontogeny

Here we discuss two aspects of animal ontogeny related to grazing personalities. Firstly, the ontogeny itself and the changes in behavior observed in animals over their lifetime. Grazing personalities are consistently observed across situations and over time. However, the behavior of an animal changes along its behavioral development or maturation. For example, Van Moorter et al. (64) conducted a study at contrasting locations in France to compare the exploration behavior of yearling (8–15 months old) roe deer (*Capreolus capreolus*) prior to the settlement phase of dispersal against the exploration of adult individuals (>2 year old). Young roe deer had larger exploration behavior than adult deer. The results proved that yearling roe deer leave their natal home range and display a period of exploration in spring and summer as part of their natural maturation process. Adult individuals settle down later in life and explore smaller areas. The example above shows that individuals display changes in grazing behaviors along their ontogenic development. Furthermore, within behavioral development phases, behavioral differences among animals are maintained from early life and along their lifetime. Finally, behavioral differences among individuals detected early in life can be used as predictors for divergent grazing personalities displayed at mature life phases.

The second aspect regarding animal ontogeny affecting grazing personalities is the importance of environments and emotional states experienced early in life (including experiences of predecessors in preconception) to influence the gene expression of behavioral and personality traits. Maternal effects early in life that induce changes in gene expression and thereby of phenotypic behavior have been documented in birds. For example, wild females of the altricial canary (*Serinus domesticus*) regulates the use of androgens when laying eggs in a way that late born chicks have higher levels of testosterone (65). Thus, chicks from late laid eggs showed faster embryonic development, increased muscular development and more begging behavior than chicks of early laid eggs. All these traits made the younger chicks of the clutch to be more competitive than older (i.e., earlier born) chicks. Different hormonal environment experienced early in life can induce changes in the expression of genes controlling physiological and behavioral traits, conferring a social hierarchy, which is maintained later during adulthood (65). To our knowledge, no study had documented changes of gene expression due to early-in-life experiences in large herbivores [but see study of Candemir et al. (66) with mice].

In the following paragraphs, we explain and exemplify how adaptive responses to early life experiences are determinant in shaping the gene expression of an individual and how such responses can be inherited epigenetically.

#### Epigenetics

The epigenetic inheritance system of the GP-model is a set of mechanisms that modifies DNA arrangement and that affects the expression of genes related to grazing personalities without causing alterations to the nucleotide sequence. Epigenetic mechanisms stimulate, discourage, or inhibit the expression of genes through DNA folding and transcriptional activities. Most known epigenetic mechanisms are DNA methylation and histone alterations (67). Such mechanisms mediate the interface between the genomic control over grazing behaviors, and responses to stimuli such as the social and biophysical environments and the emotional state of animals. Adaptive and maladaptive responses to stimuli are reflected in the phenotypic grazing personality of individuals that undergo changes to their epigenetic state and thus modulate their gene expression. Thus, alterable epigenomes—i.e., facilitated epigenotypes (probabilistically controlled by the genotype) and pure epigenotypes (not controlled by the genotype outside the affected locus)—depend on stimuli signals and is modified according to each individual's experiences (68). The transgenerational epigenetic inheritance is the transference to offspring and following generations of adopted epigenetic states in response to stimuli. Steroid hormones mediate a particular case of epigenetic modifications in response to stress (68). The study conducted by Howery et al. (2) in an extensive grazing allotment in Idaho (USA) reported that the majority of individual beef cows (78%) showed high-fidelity to home range and habitats, returning to these feeding areas in consecutive years. The study was carried on for another 4 years (1990–1993) to test if offspring and cross-fostered offspring maintained fidelity to the home range and habitats where they were reared and whether grazing behavior of dams and foster dams influenced their grazing behavior (36). These authors reported that home range and habitat fidelity was displayed by dams and foster dams as well as by yearlings and cross-fostered yearlings. They concluded that grazing behaviors experienced early in life conditioned the behavior in adulthood, and this was observed independently from yearlings being reared by their dams or by foster dams. Habitat fidelity decreased however with a severed drought and in response to the grazing behavior of other peers. These studies showed that grazing behavior was consistent over time and it was transferred to the progeny and foster-progeny. While parental effects of dams and foster-dams were corroborated, at that time, genetic heritability of grazing behavior was not tested and remained unknown. The grazing behavior of dams and yearlings was affected by a severe drought in 1992, which illustrates the plasticity of grazing behaviors responding to changing biophysical environment. Parental effects and peer effects modulated the grazing patterns of yearlings accordingly to the social herd environment experienced early and in subsequent stages of life. Howery and Bailey (43) attributed these results to a combination of nature (genetic) and nurture (learned), although, the latter could also be attributed to epigenetic inheritance. In the following section, we present examples showing how the emotional state of animals can induce changes on the expression of behavioral genes.

#### Emotional Operating System

Conscious and unconscious internal states of the brain dictate the mental well-being of mammals. While fulfilling their physiological needs, animals can react to external and internal stimuli to attempt to minimize negative emotions and to seek positive emotions (69). For example, grazing actions and reactions of ruminants and foragers in general are conditioned by their current emotional state, past experiences, and expectations (70, 71); referred to as cognitive mechanisms in the GP-model. Emotions modulate the expression of grazing genes through epigenetic states (inheritable emotional states) and/or affect the observed grazing behaviors directly (i.e., see the two arrows of emotional state in Figure 1). For instance, domestic chickens (*Gallus gallus*), under a social environment of intermittent isolation early in life developed a lowered response to corticosterone, which restrained stress (72). Using microarrays immediately after the treatment, treated chickens have upregulated the function of genes related to stress. Later in life, chickens treated with social isolation displayed a decreased reactivity of the hypothalamic-pituitary-adrenal axis, increased growth and improved associative learning in comparison with untreated chickens. The study provided evidence of transgenerational inheritance triggered by the chickens' emotional state. The emotions and the emotional state of animals affected their immediate behaviors; also experiences early in life might have underpin lifetime “conditioning” that altered the epigenetic environment of specific genes. Such effect was transferred to the progeny. Negative and positive emotions may affect (non-heritable) and modulate (heritable) the behavior of animals. For example, among these emotions, stress has been studied extensively because of the relevance to animal welfare, health and fitness. As individual animals display different coping styles while facing stressful situations, their emotions, emotional state, and ultimately their welfare, depends upon their individual personalities (46).

### Grazing Traits of Individuals

On the one hand, quantitative and continuous traits are commonly used to describe grazing behaviors along continuum gradients (28, 32). On the other, grazing personalities as categorical attributes of consistent behaviors may emerge because of the existence of trade-offs among correlated traits. Thus, animals may adopt contrasting strategies (52) such as the contrasting proactive and reactive personalities, *sensu* “life-history theory” (51) or the fast and slow metabolisms, *sensu* “pace-of-life syndromes” (73).

Behavioral studies on foraging animals are commonly limited to describe two types of grazing animals, which account for the extreme behaviors observed at the opposite ends of a continuum axis. For example, the residual feed intake was estimated for nearly two thousand dairy cows and a continuous gradient of this parameter was obtained. Then, individuals displaying the lowest and highest residual feed intake within this gradient were selected for further research [i.e., 183 and 16 selected individuals (74) and (9), respectively]. Similarly, animals of several species have been classified into two contrasting types (Table 1). For example, ruminants have been categorized as either riparian or uplands users (2, 36), bold or shy explorers (48, 49), bottom-dwellers or hill-climbers (1, 32, 40, 41).

Alternatively, a diverse range of discrete personalities can be depicted by integrating multiple behavioral “dimensions” (e.g., grazing traits) to describe and classify animals that show distinctive behaviors (17, 75). A multi-dimensional approach applied to grazing behaviors allows the conceptualization (and description) of consistent movement patterns both within species and across species. For example, studies have investigated a large diversity of foraging species and thus clustered individuals into four major types of so-called movement syndromes (25), movement strategies (26), or functional movement classes (27). These studies included, thirteen species of several vertebrate taxa of herbivores and carnivores (25); large herbivores such as the African elephant (*Loxodonta africana*), giant Galapagos tortoise (*Chelonoidis* spp.), and mule deer (*Odocoileus hemionus*) (26); and 92 species of marine life with feeding habits of carnivorous, zooplankton and algae feeders (27). The four movement types of these three studies were described and similarly named as: centered home-range, territorialists, nomads, and migrants under movement syndromes (25); as resident, multi-patch, nomadic and migration under movement strategies (26); and as resident, occasional, irruptor, and roamer under functional movement classes (27). The studies found four common movement patterns across several taxa that have different modes of movement (e.g., terrestrial locomotion, swimming, flying) and different feeding habits. For example, there were herbivores [e.g., African elephant, plains zebra (*Equus quagga*), springbok (*Antidorcas marsupialis*), mule deer, and several algae feeding marine species] and carnivores [e.g., African wild dog (*Lycaon pictus*) and several fish feeding marine species]. Furthermore, the authors observed these common movement patterns consistently across situations and over time, a condition for behavioral personalities. We anticipate that grazing ruminants and other large herbivores consistently display such common grazing patterns within herds, populations and species and even across species (i.e., regardless of species identity).

Finally, another alternative would be if grazing patterns and behaviors of grazing ruminants and other large herbivores are displayed as normally distributed variables and genetically independent traits that show no phenotypic correlations (37, 76). In such a case, conceptualizing categorical grazing personalities might be challenging or even inappropriate.

## Collective Grazing Personalities

“*The social environment and interactions have a lifelong influence on what an animal eats and where it goes […]. In herbivores, social organization leads to culture, which is the collective knowledge and habits acquired and passed from generation to generation about how to survive in a particular environment”* (77).

In this section, we focused on the collective grazing personalities of the GP-model (Figure 1, right side): from collective genotypes (at the top), through regulatory systems modulating plastic responses (middle), to phenotypic grazing personalities as observed in herds of grazing ruminants and other large herbivores (at the bottom). In section Gene Pools and Allele Frequencies, we hypothesize that the allelic variation and frequency of grazing genes determine the emergence of grazing personalities at collective level. Section The Social Environment of the Herd presents the collective social environment as the main regulatory system that shapes grazing personalities at collective level. In section Grazing Traits of Herds, we present examples and discuss the emergence of collective grazing patterns as consistently observed across contexts and over time.

### Gene Pools and Allele Frequencies

The existence of distinctive grazing personalities among individuals and the coexistence of divergent personalities within populations (so-called behavioral polymorphic populations) are both products of evolutionary processes. Selection acts over phenotype through differential fitness (e.g., individuals achieving different rates of survival and reproduction), which is then reflected in the gene pool of the group (12, 51). Animals that achieve longer lives, and/or greater reproduction rates under certain social and biophysical conditions, will produce more offspring. In this way, outperforming phenotypes with greater fitness get larger representation within the herd, making their alleles more common in the gene pool. Inversely, phenotypes with lower fitness are less represented in the population and in turn, their alleles become less common. Changes in social or biophysical environments may affect the fitness of distinct grazing personalities and lead, over generations, to changes in the allele frequencies of genes. Despite their lower performance, low fitness phenotypes still reproduce and therefore, their genes are maintained (78). Mating success of behaviorally distinct individuals would influences the allele frequencies of the population. Populations may have different behavioral morphs that exist at specific ratios. Here are two examples.

Lampert et al. (79) reported genetic associations with divergent behavioral strategies of mating in panuco swordtail fish (*Xiphophorus nigrensis*). Divergent mating-strategy and morphs of panuco swordtail fish are genetically associated with specific alleles and therefore, these populations seems to be genetically and phenotypically polymorphic. The small male morphs have relatively smaller swords, have a female appearance and are less ornate than large males, which are gifted with larger swordtails and are much more decorated. Females prefer mating with large males, which are territorial and court them. The apparent reproductive disadvantage of small swordtail fish morphs does not stop them mating, and instead of undertaking courtship, small males chase and force females to copulate. By adopting a different behavioral mating strategy, small fish morphs successfully passes their genes ensuring the persistence of this morpho-behavioral phenotype. In the second example, Pruitt and Goodnight (80) reported that natural populations of communal spider (*Anelosimus studiosus*) have behavioral polymorphic individuals labeled as aggressive and docile. Populations of spiders growing under contrasting environmental conditions such as high and low availability of resources have different ratios of the aggressive to docile phenotypes. The phenotype ratio largely explained the reproductive success of the colony and determined the behavioral attributes of the colony. The authors concluded that aggressive:docile behavioral ratio would ensure long-term survival at the collective level. The phenotype ratio was site-specific and was the result of a collective-driven selection. On artificially made populations, switches of the phenotype ratio toward the ratio of spiders' origin (and regardless of the environmental conditions i.e., maladaptive responses) can be attributed to collectively controlled inheritance.

To our knowledge, there have not been any studies looking at genotypic diversity, composition and relative frequency of grazing genes in ruminant herds. Since the very beginning of animal domestication, herders are selecting individual animals by their behavior (e.g., docility). But it is only in the last 30 years that scientists started to recommend culling individual animals that display undesired grazing patterns (2, 81). Certainly, the behavioral selection conducted in the past over domesticated herbivores has shaped the gene pools of present-day herds. However, it is unknown how this selection has affected their grazing patterns. Similarly, environmental changes, such as fragmentation of natural ecosystems, limited animal migration or selective hunting, has affected the gene pools and relative frequency of grazing genes of herds of wild animals and in that way, may have modified their collective grazing personalities. This has been exemplified by the selective capturing of fish with nets over wild fish populations (24). As seen with the artificially-made colonies of communal spiders (80), we speculate that the ratio of genotypic grazing personalities within a herd of ruminants might be regulated collectively to ensure long-term survival of the group. As the ratio of genotypes within a herd might be site specific, it is possible to speculate that such collective traits are inherited epigenetically.

The recent discovery of nucleotide variation in grazing genes and their association with the grazing patterns of individual animals opens the opportunity to search for an ideal grazer; one that displays the “best” grazing personality (32, 40–42). However, large herbivores do not graze alone but in herds of interacting animals, where individuals display a range of distinct grazing personalities that shapes the grazing personality of the herd. In this way, herds have unique attributes of grazing behavior (see section Grazing Traits of Herds). At collective level, genetically similar herds may display different personalities because of the plastic expressions of grazing patterns. This is discussed in the following section.

### The Social Environment of the Herd

The interactions among conspecifics constitutes the social environment of herds. Such interactions establish the social status occupied and the behaviors adopted by each individual. For example, the roles of leader and follower (45), dominant and submissive (38), and producer and scrounger (82), are extensively documented in ruminants, birds and other foraging species. Socially responsive individuals adjust their behaviors according to the social context and within the limits of their personality plasticity (83). Thus, the social herd environment is a major factor of behavioral variation that affects the phenotypic expression of grazing personality and its plasticity at the individual and collective level (61). In section The Effect of Personality Plasticity and Regulatory Systems on Grazing Patterns, we provided examples of how the social environment (e.g., social isolation and parental care) affects the behavior of individuals. Similarly, the emergence of socially central individuals (e.g., leader and dominant animals) conditions collective grazing behaviors. For example, in Highland cattle (*Bos taurus*), Sueur et al. (45) reported that castrated mature males provided leadership and promoted group cohesiveness to juvenile cattle. These authors suggested using trained matured castrated males to increase grazing intensity of targeted areas. In another experiment with groups of fallow deer (*Dama dama*), Stutz et al. (84) showed that high aggregation and cohesiveness working toward increasing safety against predators have reduced the individual and collective exploitation of preferred and more nutritious diets. Thus, the collective perceived risk of predators influences collective exploration and utilization of feed sources. Another way to study the effects of collective behaviors is by replacing (or removing) socially central individuals. Vital and Martins (85) removed the key individuals from a group of zebrafish (*Danio rerio*) and reported reduced learning of foraging skills. In bottlenose dolphins (*Tursiops truncatus*) the presence of certain individuals was crucial to maintain interactions between subgroups (86). However, in beef cattle the effects of socially central individuals might be only relevant in small size herds, for example <40 individuals, where the fidelity of individuals to the group they belong to is relatively high; on the contrary, social bonds in larger herds are expected to be weaker (87, 88). In the collective context of colony living organisms, the social environment is crucial for the survival and fitness of the group as well as for the relative success of each individual (33); to a certain extent, this is also the case for collective grazers such as grazing ruminants and other large herbivores.

The GP-model establishes that stimuli from the social and biophysical environments and the emotional state of animal affects the displayed grazing personalities of individuals, which in turn are transferred to the grazing patterns displayed collectively (see section The Effect of Personality Plasticity and Regulatory Systems on Grazing Patterns). Similarly to the case of individuals, the social environment of the herd might influence the gene expression of collective grazing traits and therefore modulate the phenotypic grazing personalities as observed collectively. However, until now, it is unknown whether there are genes controlling collective behavioral traits in ruminants and, if so, whether the social environment controls its expression. A combination of social learning and a segregation of leader and followers could also explain collective behaviors (85). We posed these unresolved aspects using question marks in the GP-model (Figure 1). In the next section, however, there are examples of grazing traits measured at collective level.

### Grazing Traits of Herds

Based on behavioral genetics, Gross (89) described three main pathways to explain phenotypic polymorphism of behavior displayed by individuals within animal populations. Firstly, the so-called “alternative pathway” which considers a frequency-dependent selection of animals that maintains genetically polymorphic populations with individuals displaying behavioral polymorphism and achieving similar fitness. Secondly, the “mixed pathway” occurs in genetically monomorphic populations with individuals displaying mixed behavioral tactics. Finally, the so-called “conditional pathway” occurring in genetically monomorphic populations where individuals display a set of behavioral tactics according to state-dependent conditioning. For the GP-model and for any study of grazing herds in general, it is crucial to bear in mind that herds of ruminants are phenotypically behaviorally polymorphic. Within a herd of ruminants, individuals coexist displaying a range of distinctive grazing personalities. While the alternative pathway attributes the phenotypic behavioral polymorphism to genotype variation (i.e., personality genotype in the GP-model), mixed pathways and conditional pathways apply to populations comprised by genetically monomorphic individuals. As previously presented in section The Effect of Personality Plasticity and Regulatory Systems on Grazing Patterns, the personality plasticity at collective level accounts for the variable gene expression and therefore, different phenotypic outcomes from genetically identical individuals may take place. We hypothesize that the mixed pathway may correspond to variations attributable to the epigenetic system (heritable), and that the conditional pathways may correspond to direct effects over the emotional state. For the previous, adopted behaviors might be transferred to offspring and therefore show transgenerational epigenetic inheritance; for the latter, behavioral polymorphism may be observable only in the animals that adopted such behavior as a direct response to their emotional states.

We set the GP-model using an individual-based approach of grazing personalities to explain distributional grazing patterns as observed in real herds of ruminants. Gueron et al. (47) presented a model that simulated distributional patterns of grazing herds based on a set of behavioral traits that were applied to individual agents. The authors applied a hierarchical decision-making algorithm, with rules-of-thumb establishing individual sensitiveness to crowding and attraction to conspecifics that applied respectively according to a repulsion zone (animals getting too close), an attraction zone (animals getting too far), and an intermediate buffer zone called neutral zone without response. Simulations were ran for a thousand time-steps of individuals that displayed different behavioral traits, such as walking speed and sense of orientation toward a targeted direction. Gueron's model showed differences in herds distribution and fragmentation as it happens in real herds. The model showed that integrating behavioral, physiological and individual decision-making traits could reproduce attributes of interacting “grazing” animals. From individual differences in grazing traits emerged collective behaviors of herd fragmentation and distributional patterns.

Gueron's mechanistic simulations were later tested and validated in a similar model using groups of sheep of variable number (two, four, six, or eight sheep) of either exclusively bold individuals or exclusively shy individuals (90). In support of individual-based approaches, the findings of these authors showed that the grazing patterns observed in interacting animals derive from individual behavioral traits and interaction rules; however, behavioral traits at the group level, such as the strength of social attraction, seems to control emergent decision-making mechanisms at collective level. A further step on the simulation of grazing herds was achieved by Spiegel et al. (52). These authors simulated grazing agents with divergent movement traits in variable contexts of vegetation patchiness. With some similarity to the simulations done by Gueron et al. (47), Spiegel et al. allocated divergent behavioral traits to groups of individuals “grazing” along increasing levels of vegetation patchiness, i.e., from low patchiness where pixels of nine different vegetation resources were uniformly mixed (patch size equals pixel size), through medium patchiness with randomly mixed pixels (mid-size patches), to high patchiness where pixels of each resource are highly aggregated forming large and discrete vegetation patches. Comparing divergent personalities such as slow and fast explorers, these authors concluded that under low patchiness, fast explorers would achieve higher foraging efficiency than slow explorers. This would be reversed, however, in grazing lands with discrete vegetation patches. Such results are consistent with real experiments in dairy cows (9). Spiegel's et al. (52) scenarios showed that seasonal dynamics of vegetation would alternatively benefit one or another grazing personality at different times of the year, highlighting the temporal variation of animal performance in support of the existence of herds with behavioral polymorphism. Finally, these authors pointed out the emergence of a complex group-level structure displaying collective grazing patterns with its own attributes (e.g., clustering of similar phenotypes, home range size, and structure), which changed along environmental gradients (e.g., vegetation patchiness). Interestingly, individual-based simulation models set behavioral rules and traits to be repeated over time [i.e., 1,000 and 2,000 time steps in Gueron et al. (47) and Spiegel et al. (52), respectively] and even across different contexts such as a gradient of vegetation patchiness (52). By allocating different values of behavioral traits to individuals that coexist and interact with each other, simulation models recreated real ruminant herds as mixed behaviors displayed consistently over time and across situations; therefore complying with conditions of grazing personalities used in the GP-model.

Individuals displaying divergent personalities comprise herds of ruminants, which are recognized and described as extended families that maintain cohesiveness and display unique identities (77). So, how can we characterize and compare the unique identities of ruminant herds (i.e., collective grazing personalities)?

One way to value behaviors at collective level is by using grazing traits measured in individuals while performing within the herd and by integrating these individual values into an averaged and/or weighted value. Additionally, the statistical dispersion of behavioral traits (e.g., coefficient of variance) within herds can be used for comparisons among herds. To our knowledge, there are not many studies with such examples. Partially, this might be because of the challenge of measuring grazing behaviors in all members of the herd while grazing as a herd. However, this might be also because of the lack of conceptualizing collective measurements of grazing behaviors, although, this has been proposed for other social living animals such as foraging insects (33). Sueur et al. (45) studied leadership within four Highland cattle groups (groups ranging from 8 to 21 individuals), but did not compare collective behaviors among groups. Rudin et al. (91) compared behavioral traits on two groups of over 500 Australian field crickets (*Telogryllus oceanicus*) growing under contrasting social environments of “silent” or “signing” individuals. Based on statistical differences in the mean value and standard error on distance traveled and speed measured in individuals, these authors concluded that the social environment significantly affected “the repeatable aspect of behavior (i.e., personality),” and that behavioral changes were heritable. However, Rudin et al. (91) measured traits in individuals pulled apart from the group rather than on individuals performing within a group. Several studies in the past compared distinct behaviors displayed in ruminants (9, 32, 39) and authors commonly conclude that “individuals” pertaining to a certain group behave differently to “individuals” pertaining to another group rather than assessing collective behaviors. We advocate for comparisons of different groups that display collective grazing personalities with their unique attributes.

Another way applicable to certain scenarios and for certain traits is by representing collective grazing behaviors with the behavior of one or a few animals of the herd. For example, Liao et al. (28) studied the grazing behavior of 20 herds of beef cattle in five different study sites of Southern Ethiopia. These authors derived collective behavioral traits such as daily allocation of time to travel, grazing, and resting by averaging the behavior monitored in three cows of each herd with GPS collars. Pastoral people herded their animals to daily foraging areas and brought them back to their camps for overnight. The herd was moved as a relatively compact group, thus, monitoring of any three cows of each herd would be sufficient to provide comparative information among herds. These authors reported different daily patterns of grazing behavior of monitored herds and provided insights on the different foraging habitats used by different herds with details on greenness, elevation and terrain slope.

Here, we mention attributes of ruminant herds and grazing traits relevant to collective grazing personalities. For example, home range was defined as the spatial expression of behaviors [that individual] animals perform to survive and reproduce (92) in a defined timescale (93). Thus, a certain number of individuals that comprise a herd occupies, needs or is allocated to an area with features of size, shape and biophysical conditions. Similarly, one could compute the area utilized by a herd, for example, on a daily basis. Fragmentation (47), cohesiveness (94) and assortativity (52, 95) are examples of group-level traits that in a future can be used to study collective and individual grazing attributes as well as the impact of grazing herds to ecosystem functions of grazing lands or to animal welfare. For example, Foister et al. (96) used phenotypic attributes of social interactions measured at group-level (i.e., social network properties) to predict consistent aggressive events (i.e., a personality dimension) among pigs reared as a group in pens. In beef cattle, the centrality of individuals as a specific collective measurement rather than the number of individuals determined the group composition and affected the social stability and stress of the herd (97).

## Illustrations and Implications

Figure 1 presents a hypothetical example with individuals (left side) differing in the allelic variations of two grazing genes, which comprise a herd of ruminants (right side). Grazing personality pathways between an individual's genotype (I_x_) and its displayed grazing personality (iGP_x_) involve several intermediate and concatenated traits, which have a regulatory system of the gene expression. Following the GP-model, we described this example starting on the individual genotype (top left), going through stimuli that influence the expression of grazing genes (middle left) to yield in the phenotypic grazing personality of individual grazers (bottom left). As ruminants graze in herds, individual genotypes were aggregated into the collective gene pool (top right), then, we discuss the modulation of the gene expression at group level (middle right) and finally describe the collective personality of the herd.

### Grazing Personality Genotype

Individuals with allelic variations I_1_ and I_2_ display shy grazing personalities named iGP_1_ and iGP_2_, respectively. Individuals with shy personalities occupy relatively small home ranges, stay at relatively short distances from one another and prefer grazing flat terrain in low altitude habitats. As personalities are phenotypically plastic, under certain conditions, iGP_1_ and iGP_2_ cannot be differentiated because of phenotypic overlap. Individuals with allelic variation I_3_ are associated with animals displaying a bold grazing personality named iGP_3_. Such herbivores show relatively large home ranges, they graze alone or at relatively large distance from one another and show grazing preference for steep slope terrain in high altitude habitats. Regardless of conditions, IGP_3_ always display discernible grazing patterns from the previously described personalities. For example, iGP_1_ and iGP_2_ could be similar to bottom dweller cattle and, iGP_3_ to hill climber cattle, which display divergent indexes of landscape use and exhibit divergent grazing patterns (1). These cattle have genetic associations to gene markers overlaying the glutamate receptor 5 (*GRM5*) gene and the mastermind-like 3 (*MAML3*) gene (32). In the example, these genes are represented with hexagon and triangle shapes in Figure 1. For simplicity, only two of the five genes reported by Bailey et al. (32) are represented in the GP-model. Applying individual-based models, grazing patterns of herbivores can be simulated by using traits such as walking speed and sense of direction toward a preferred habitat and by applying variable responses to stimuli such as to vegetation patchiness, like variable walking acceleration or proximity to conspecifics (47, 52, 90). In our example, iGP_1_ and iGP_2_ have equal allelic variation as I_1_ *GRM5*[A] = I_2_ *GRM5*[A]. This genotype determines animals to have low concentrations of blood cortisol that makes them to display low walking speed and travel relatively short distances (39, 98, 99). For this example, we establish that *GRM5*[A] animals prefer grazing in flat terrains. Walking acceleration and attraction zone to conspecifics are also similar (iGP_1_ ~ iGP_2_) making them quickly accelerate toward conspecifics that get away and to do so at relatively short distances. These personalities differ in their allelic variation I_1_ *MAML3*[T] ≠ I_2_ *MAML3*[G], responsible of sense of orientation toward preferred areas. For example, *MAML3*[T] animals display a high sense of orientation and *MAML3*[G] express a low sense of orientation (iGP_1_ > iGP_2_). I_3_ animals differ from both previous genotypes by having *GRM5*[G], which is phenotypically expressed with a high blood cortisol concentration. *GRM5*[G] animals display fast walking speed, and therefore I_3_ animals travel relatively long distances. For this example, we establish that *GRM5*[G] animals prefer grazing in steep slope terrain in high altitudes. iGP_3_ walking acceleration is low and attraction zone to conspecifics is long, therefore, iGP_3_ individuals accelerate slowly toward conspecifics that get away and do so when conspecifics are relatively far away. iGP_3_ has equal allelic variation to iGP_2_ animals for the sense of orientation trait (I_2_ *MAML3*[G] = I_3_ *MAML3*[G]), therefore show low sense of orientation toward its preferred mountainous terrain.

In a herd of ruminants, allelic diversity is defined as the number of different alleles of a grazing gene present when accounting for all individuals. Allelic composition refers to which alleles in particular are represented. Finally, relative allelic frequency refers to the proportion of each allelic variant of grazing genes. While these two previous attributes do not necessarily depend of the number of members but on their genotype, the latter, depends on combining the genotype of members and their proportional representation. Finally, the total size of the herd, at equal proportion of individual grazing personalities, affects the collective personality (not considered in this example). In our example in Figure 1, two grazing genes, *GRM5* and *MAML3*, are shown in three grazing personalities I_1_, I_2_, and I_3_ that comprise herd one (H_1_). Each gene has two variants. Therefore, the allelic diversity for either of these genes in H_1_ is two. The allelic composition of *GRM5* is Adenine and Guanine, while for *MAML3* is Thymine and Guanine. Note that the total existing allelic variation for these genes is much larger than in our example; Bailey et al. (32) reported four possible nucleobases (adenine, cytosine, thymine and guanine) at six different positions in *GRM5*, and the nucleobases thymine and guanine for *MAML3*. In Figure 1, we did not specify the number of individuals of each genotype nor total number of individuals comprising the herd. However, we represented the relative allelic frequency of grazing genes *GRM5* and *MAML3* establishing equal number of individuals (n = 10) of each genotype. For example:

If I_1_ *n* = 10; I_2_ *n* = 10; I_3_ *n* = 10, then the relative allelic frequency in H_1_ would be: *GRM5* x2[A]: 1x[G]; *MAML3* x1[T]: x2[G].

### Personality Plasticity

Despite the genetic determination of cortisol concentrations in blood in individual animals, it has also been revealed that its expression is affected by stimuli, such as during experiments of social isolation [see Goerlich et al. (72) in section The Effect of Personality Plasticity and Regulatory Systems on Grazing Patterns]. For example, the use of low-stress herding techniques might reduce cortisol concentration in the blood of ruminants and foster the use of targeted areas because of emotional state of lower predation risk as in comparison with animals under “traditional” herding techniques (100). In our example, reduction of the concentrantion of cortisol in blood is established to reduce walking speed and also daily traveled distance. We represented personality plasticity on the phenotype of the hypothetical individuals. In Figure 1, iGP_1_ and iGP_2_ overlap each other and under certain conditions it will not be possible to distinguish them by simple phenotypic observation. On the other end, iGP3 is separated toward the right of the GP-model and representing therefore that differences in grazing personalities are phenotypically observable.

### Grazing Personality Phenotype

The GP-model as shown in Figure 1 represents genetically polymorphic individuals (i.e., individuals with different alleles) that comprise the collective gene pool and relative allelic frequency of grazing genes of a herd. Phenotypically, in such a herd coexist individuals that display distinct grazing personalities. iGP_1_ and iGP_2_ individual display slow walking speed and travel short distances. As soon as conspecifics move away a relatively short distance, these individuals will accelerate and reduce distance to conspecifics. These grazing personalities prefer flat and low altitude habitat, where they graze more intensively and spend more time than on steep slopes located in high altitude habitats. iGP_1_ individuals will return quicker and more often to vegetation patches of their preferred habitat than iGP_2_, because of the lower sense of orientation of the latter. Therefore, iGP_1_ tends to utilize its preferred habitat for a longer time. Herds comprised purely of either iGP_1_ or iGP_2_ individuals are less fragmented, move slowly and have smaller home ranges [slow-explorer *sensu* (52)]. In grazing lands where patches of vegetation are small and homogeneously distributed, these two personalities may display similar grazing patterns (i.e., phenotypically similar) because the sense of orientation would not make a difference in distribution where non-conspicuous patches of vegetation exist. In grazing lands where significantly big patches of vegetation are heterogeneously distributed, iGP_1_ will utilize more intensively its preferred habitat, taking advantage of its better sense of orientation in comparison with iGP_2_ individuals (i.e., phenotypically dissimilar). Herds comprised purely of iGP_3_ individuals are highly fragmented, move faster, and individuals graze at greater distances from one another. iGP_3_ individuals graze alone or in relatively small groups that occupy larger home ranges than iGP_1_ or iGP_2_ individuals. iGP_3_ individuals prefer steep slope areas in high altitude habitats and have low sense of orientation. As per their low sense of orientation, these animals will show similar grazing patterns in homogeneous and heterogeneous grazing lands. However, iGP_3_ are "always" phenotypically dissimilar to the other grazing personalities.

### Implications

The GP-model proposes a novel understanding of social foragers: grazing is a social activity performed by herds of interacting ruminants that display collective grazing personalities with their own unique attributes. Individuals that display distinct grazing personalities comprise behavioral polymorphic herds of ruminants. Grazing personalities of ruminants are controlled by their genetic composition and are modulated by their epigenetic states in response to the social herd environment, biophysical environment and the emotional state. Adaptive and inheritable epigenetic states confers plasticity to grazing personalities at individual and collective levels.

#### Selecting for Grazing Personality

Farmers, ranch managers and breeders may adopt the concept of grazing personalities and select for animals according to the desired and needed distinctive behaviors. By so doing, we forecast a genetic gain on herds to address major challenges faced by the pastoral livestock production industry. The identification of grazing personality genotypes and the development of the corresponding genetic markers can be used to determine the grazing personality composition of herds and to further assist in applying goal-oriented selection of animals using a relatively simple and inexpensive genetic test such as single-strand conformation polymorphism (SSCP) (101, 102).

#### Enhancing the Expression of Grazing Personalities

The GP-model establishes that grazing personalities of ruminants and other large herbivores are plastically displayed in response to stimuli (e.g., social herd environment, biophysical environment, and animals' emotional states). Such responses might be adopted and shown for the entire lifespan of animals and, can be farther transferred to their progeny through transgenerational epigenetic inheritance. This is particularly relevant for experiences occurred early in life. Exposing grazing ruminants and other large herbivores to the biophysical environmental conditions where they are targeted to perform may trigger epigenetic mechanisms and regulatory systems that foster the expression of grazing genes toward desired behaviors of individual grazers and herds. As per the GP-model, the social context in which an animal and its predecessor grow (i.e., the social herd environment) modulates the expression of grazing genes and therefore the displayed grazing personalities. For example, social environments of isolation, crowdedness, threats and fearfulness, as well as the aggressiveness of herds, affect the emotional states and modulates the individual and collective grazing behaviors and associated decision-making. Similarly, the biophysical environment might shape the expression of grazing genes.

#### Influencing Grazing Personalities Through Emotions

Grazing management practices such as fasting, supplementation or herding techniques alter animal internal states (e.g., hunger, emotions), influence animal decision-making and ultimately, modify their grazing patterns.

#### Designing Behavior-Customized Herds

The composition and relative frequency of grazing personalities of domesticated ruminant herds has been manipulated and shaped for millennia to produce docile and manageable individuals and herds suitable for living alongside and under management of humans. The GP-model proposes to apply behavioral-based selection for the design of ruminant herds matching the spatial diversity and the temporal variety of forages, foodscapes and landscapes. Pastoral livestock production systems are heterogeneous in space and time. Despite efforts to create “simple and homogeneous” systems, individualities and collective attributes of grazing patterns emerge. Herds are comprised of a mix of individuals displaying distinctive grazing personalities. Therefore, grazing patterns of ruminant herds can be manipulated through designing and deciding the relative frequency of individual grazing personalities along with the adoption of grazing management practices that foster the desired behaviors.

## Conclusions

The GP-model proposes that genetic effects (allele diversity, composition and relative frequency) and epigenetic modulation (via regulatory systems that modulate the gene expression) conditions grazing behaviors of ruminants and other large herbivores, so that, animals display grazing personalities at individual and collective levels. The interactions with the social herd environment and the biophysical environment shape the phenotypic grazing personalities of individuals. Collective grazing personalities emerge from the social interaction of individuals and their grazing personalities. The social herd environment mediates between the individual and the collective grazing personalities. This is because interacting individuals constitute the herd and create its environment. In turn, the social herd environment influences both, the grazing personality of individuals and the grazing personality of the herd.

The allelic composition and the relative frequency of grazing genes characterize the collective genotypic of grazing personalities and, therefore, there is the opportunity to develop breeding programs aiming to influence grazing patterns of ruminant herds applying behavioral selection. Because of the genetic basis of grazing behavior, animal selection maybe a useful tool to improve grazing distribution of habitat-heterogeneous livestock systems.

The displayed grazing personality of herds of ruminants and other large herbivores results from their genome and the personality plasticity. Grazing management, herding techniques, feeding strategies, and rearing practices that affect animal welfare and the gene expression of grazing traits have the potential to foster desirable grazing personalities. Managers that account for the variety of individual grazing personalities naturally displayed in ruminants, and that manipulate its proportion, can enhance ecosystem services and improve animal welfare while maintaining the productivity of livestock production systems.

The grazing personality model presented here further develops our understanding of the distribution of ruminants and large herbivores by integrating discoveries from the past few decades into models of grazing distribution and behavior (29, 30). The GP-model was inspired from and supported with scientific works conducted with a diverse range of taxa from the animal kingdom, namely bees, birds, marine species, large herbivores, ruminants, and other ungulates. Future research on grazing personalities at the individual and collective levels may confirm the hypotheses posed in the “grazing personality model” and thus contribute to a better understanding of livestock production systems, grassland science and animal behavior.

## Author Contributions

CM wrote the manuscript. CM, PG, TM, and JH made substantial, direct and intellectual contribution to the work, and approved it for publication.

### Conflict of Interest

The authors declare that the research was conducted in the absence of any commercial or financial relationships that could be construed as a potential conflict of interest.
